# The influence of gut microbiota on the progression of Type 2 Diabetes: a new perspective for treatment and prevention

**DOI:** 10.17179/excli2024-7036

**Published:** 2024-04-30

**Authors:** Lysandro P. Borges, Pamela C. de Jesus, Jessiane B. de Souza, Deise M. R. R. Silva, Pedro H. M. Moura, Ronaldy S. Santos, Marina dos S. Barreto, Adriana G. Guimarães, Lucas A. da M. Santana, Otavio Cabral-Marques, Eloia E. D. Silva

**Affiliations:** 1 Department of Immunology, Institute of Biomedical Sciences, University of São Paulo, São Paulo 05508-220, SP, Brazil; 2 Department of Pharmacy, Health and Biological Sciences Center, Federal University of Sergipe, São Cristóvão 49107-230, SE, Brazil; 3 Graduate Program in Dentistry, Health and Biological Sciences Center, Federal University of Sergipe, Aracaju 49060-102, SE, Brazil; 4 DO'R Institute for Research, São Paulo, Brazil; 5 Department of Medicine, Division of Molecular Medicine, Laboratory of Medical Investigation 29, University of São Paulo (USP) School of Medicine, São Paulo, Brazil; 6 Interunit Postgraduate Program on Bioinformatics, Institute of Mathematics and Statistics (IME), University of Sao Paulo (USP), Sao Paulo, SP, Brazil; 7 Department of Clinical and Toxicological Analyses, School of Pharmaceutical Sciences, University of São Paulo (USP), São Paulo, SP, Brazil; 8 Department of Biological Sciences, Health and Biological Sciences Center, Federal University of Sergipe, São Cristóvão 49107-230, SE, Brazil

## ⁯

The human gut microbiota, a complex community of microorganisms residing in our gastrointestinal tract, has emerged as a critical factor influencing metabolic health. Studies have increasingly shown that dysbiosis, an imbalance in these microbial populations, may contribute to the development and progression of Type 2 Diabetes (T2D) through multiple mechanisms (Scheithauer et al., 2020[5]). Firstly, gut microbiota has been implicated in the modulation of host inflammation and immune response, which are known contributors to insulin resistance (Scheithauer et al., 2020[5]). The translocation of bacterial lipopolysaccharides into the bloodstream, stemming from a compromized intestinal barrier, is thought to elicit systemic inflammation (Ghosh et al., 2020[3]). This process is supported by the observation that high-fat diets, a risk factor for T2D, can alter gut permeability and microbiota composition.

Secondly, the microbiota influences the fermentation of dietary fibers and the production of short-chain fatty acids (SCFAs), such as butyrate, propionate, and acetate (den Besten et al., 2013[1]). These SCFAs have been shown to exert beneficial effects on host metabolism by enhancing insulin sensitivity, regulating appetite, and energy expenditure (den Besten et al., 2013[1]). Hsieh et al. (2018[4]) for example, demonstrated that bacteria from the Bifidobacterium and Lactobacillus genera, recognized for their significant production of SCFAs, have a correlation with a reduction in HbA1c levels in the blood. Therefore, the manipulation of SCFA production through dietary interventions presents a promising avenue for T2D management.

Thirdly, the gut microbiome affects bile acid metabolism, with certain bacterial strains modifying bile acid profiles and signaling pathways. Altered bile acid signaling has been linked to glucose homeostasis, suggesting another potential target for therapeutic intervention (Gao et al., 2022[2]). The study by Sun et al. (2023[7]) indicated promising results on the relationship between the existence of bacteria of the genera Flavonifractor, Haemophilus, the Clostridiaceae family, the genus Actinomyces and the genus Candidatus Soleaferrea in the intestinal tract with the appearance of T2D, but the authors themselves consider the possibility that the findings are coincidental and subject to the context of the population studied. Given these insights, it becomes imperative to consider the gut microbiome as a target for T2D treatment and prevention strategies. Interventions such as personalized nutrition, prebiotic and probiotic supplementation, and even fecal microbiota transplantation should be explored for their potential to restore a healthy gut microbiota and mitigate T2D progression (Su et al., 2022[6]). 

Lastly, we advocate for future research endeavors to prioritize the identification and characterization of microbial strains and metabolites with therapeutic potential in T2D. By leveraging cutting-edge technologies, we can uncover novel biomarkers and therapeutic targets within the gut microbiota landscape. This knowledge could fuel the development of innovative microbial-based interventions tailored to address the underlying pathophysiology of T2D. This could lead to the development of microbial-based therapies that are tailored to the individual's microbiome profile. Further studies are warranted to validate and expand upon our current understandings, which could contribute to the development of a more comprehensive and globally recognized approach to precision medicine for T2D. This may ultimately lead to the development of more effective personalized treatments targeting the gut microbiota in these patients.

## Declaration

### Funding and assistance

Not applicable.

### Conflict of interest

None.

## References

[1] den Besten G, van Eunen K, Groen AK, Venema K, Reijngoud DJ, Bakker BM (2013). The role of short-chain fatty acids in the interplay between diet, gut microbiota, and host energy metabolism. J Lipid Res.

[2] Gao R, Meng X, Xue Y (2022). Bile acids-gut microbiota crosstalk contributes to the improvement of type 2 diabetes mellitus. Front Pharmacol.

[3] Ghosh SS, Wang J, Yannie PJ, Ghosh S (2020). Intestinal barrier dysfunction, LPS translocation, and disease development. J Endocr Soc.

[4] Hsieh MC, Tsai WH, Jheng YP, Su SL, Wang SY, Lin CC (2018). The beneficial effects of Lactobacillus reuteri ADR-1 or ADR-3 consumption on type 2 diabetes mellitus: a randomized, double-blinded, placebo-controlled trial. Sci Rep.

[5] Scheithauer TPM, Rampanelli E, Nieuwdorp M (2020). Gut microbiota as a trigger for metabolic inflammation in obesity and type 2 diabetes. Front Immunol.

[6] Su L, Hong Z, Zhou T (2022). Health improvements of type 2 diabetic patients through diet and diet plus fecal microbiota transplantation. Sci Rep.

[7] Sun K, Gao Y, Wu H, Huang X (2023). The causal relationship between gut microbiota and type 2 diabetes: a two-sample Mendelian randomized study. Front Public Health.

